# Comparison of amyloid chronicity and EYO in autosomal dominant Alzheimer's disease

**DOI:** 10.1002/alz.70812

**Published:** 2025-10-25

**Authors:** Julie K. Wisch, Nicole S. McKay, Matthew Zammit, Bradley T. Christian, Stephanie A. Schultz, Peter R. Millar, Natalie S. Ryan, David M. Cash, Christopher R. S. Belder, Patricio Chrem, Carlos Cruchaga, Laura Ibanez, Mathias Jucker, Igor Yakushev, Gregory S Day, Mei Murphy, Jorge Llibre‐Guerra, David Aguillon, Jee Hoon Roh, Chengjie Xiong, Guoqiao Wang, Yan Li, Suzanne E. Schindler, Cliff Jack, Eric McDade, Randall J. Bateman, Tammie L. S. Benzinger, Beau M. Ances, the Dominantly Inherited Alzheimer Networ, Tobey Betthauser, Brian Gordon

**Affiliations:** ^1^ Department of Neurology Washington University in St. Louis St. Louis Missouri USA; ^2^ Department of Radiology Washington University in St. Louis St. Louis Missouri USA; ^3^ Department of Medical Physics and Psychiatry University of Wisconsin Madison Madison Wisconsin USA; ^4^ Department of Neurology Harvard Medical School Massachusetts General Hospital Boston Massachusetts USA; ^5^ Department of Neurology University College London London UK; ^6^ Dementia Research Centre UCL Queen Square Institute of Neurology London UK; ^7^ Department of Neurology Royal Adelaide Hospital and Queen Elizabeth Hospital Adelaide SA Australia; ^8^ Adelaide Medical School University of Adelaide Adelaide SA Australia; ^9^ Centro de Memoria y Envejecimiento Fundación Favaloro – Anexo Moreno Calle Moreno Argentin; ^10^ Hope Center for Neurological Disorders Washington University in St. Louis St. Louis Missouri USA; ^11^ Department of Psychiatry Washington University in St. Louis St. Louis Missouri USA; ^12^ Hertie Institute for Clinical Brain Research University of Tübingen Tübingen Germany; ^13^ Department of Cellular Neurology German Center for Neurodegenerative Diseases (DZNE) Tübingen Germany; ^14^ Dept. of Nuclear Medicine Klinikum rechts der Isar Technical University of Munich (TUM) München Germany; ^15^ Department of Neurology Mayo Clinic Florida – Jacksonville Campus Jacksonville Florida USA; ^16^ Grupo de Neurociencias de Antioquia (GNA) Facultad de Medicina Universidad de Antioquia Sede de Investigación Universitaria (SIU) Medellín Colombia; ^17^ Departments of Physiology & Neurology College of Medicine Korea University Seoul Republic of Korea; ^18^ Department of Biostatistics Washington University in St. Louis St. Louis Missouri USA; ^19^ Department of Radiology Mayo Clinic – Mayo Building West Rochester Minnesota USA; ^20^ Wisconsin Alzheimer's Disease Research Center University of Wisconsin School of Medicine and Public Health University Hospital Madison Wisconsin USA; ^21^ Department of Medicine University of Wisconsin–Madison School of Medicine and Public Health Madison Wisconsin USA

**Keywords:** Alzheimer's disease, biomarkers, genetic causes of Alzheimer's disease, numeric methods

## Abstract

**INTRODUCTION:**

Preclinical Alzheimer's disease (AD) can be described relative to biomarker positivity onset time.

**METHODS:**

We estimated time from amyloid positivity (A+) using sampled iterative local approximation (SILA) in a longitudinal autosomal dominant AD (ADAD) sample (*N* = 379) with amyloid positron emission tomography. We compared (1) predicted age at A+ to imputed age, (2) estimated age at A+ to estimated age at symptom onset, and (3) variance in cognitive performance explained.

**RESULTS:**

Mean error between imputed and SILA‐estimated age at A+ (*N* = 26) was 1.15 years. Age at A+ explained 39% of estimated years to symptom onset (EYO) variance. Time from A+ explained 19% of cognitive composite variance and 14% of Clinical Dementia Rating Sum of Boxes CDR‐SB variance; EYO explained 43% and 57%, respectively.

**DISCUSSION:**

SILA estimates A+ age in ADAD with reasonably good accuracy. SILA‐estimated time from A+ describes the start of pathology, but the time from A+ onset to symptoms is variable in ADAD and better described by EYO.

**Highlights:**

- Amyloid chronicity predicts a 14‐year preclinical AD phase in ADAD.
- SILA accurately estimates age at A+ (MAE < 2 years).
- EYO outperforms chronicity in predicting symptom onset.
- APP mutation carriers show atypical amyloid accumulation.
- Chronicity models help reveal AD heterogeneity in preclinical stages.

## 1 BACKGROUND

Alzheimer's disease (AD) has a long asymptomatic phase that can last 20 years or more.^1^ Recent clinical trials suggest that earlier intervention is key to maximizing therapeutic response.^2^, ^3^, ^32^ Biomarkers give critical information about disease stage during the preclinical period when abnormal biomarker levels indicative of underlying pathological processes are observed in the absence of overt cognitive decline.^4^This has been demonstrated in landmark studies in late‐onset sporadic AD (sAD)^1^, ^4^, ^5^ and autosomal dominant AD (ADAD).^6^, ^7^, ^8^ Since these initial studies, several developments in modeling longitudinal biomarker trajectories have shown great promise toward understanding the timing of biomarker abnormalities during the preclinical phase of AD.

Multiple approaches have been introduced to transform biomarker levels to time course estimates of AD progression. These models are commonly based on amyloid biomarkers (e.g., amyloid positron emission tomography (PET)^9^, ^10^, ^11^ or plasma biomarkers^12^) and the assumption that these measures have a relatively uniform annualized rate of accumulation across individuals once abnormal levels are reached.^13^ From these models it is possible to generate estimates from even cross‐sectional observations as to how long an individual has been amyloid positive. This “amyloid chronicity” (i.e., amyloid time, amyloid clock) approach has been applied in sAD, with patterns being observed that are consistent with the hypothesized AD biomarker cascade,^1^ where changes occur first in amyloid biomarkers,^9^, ^10^, ^11^, ^14^, ^15^, ^16^, ^17^ followed by tau elevation in Braak stages I/II about 8 to 10 years after amyloid positivity (A+).^14^, ^15^

In contrast to sAD, wherein disease time course cannot be verified until the onset of dementia, individuals with ADAD have a more predictable time course. This phenomenon is due to the highly heritable age of symptom onset linked to specific point mutations.^18^ This allows staging by estimated years to symptom onset (EYO).^6^, ^18^, ^19^, ^20^ ADAD provides a unique opportunity to characterize the relationship between EYO and amyloid chronicity estimates. This project aims to consider amyloid chronicity as a framework for describing the preclinical and early symptomatic disease course in ADAD, with particular focus on its utility in individuals with lower cortical amyloid PET uptake. If amyloid chronicity offers reliable estimates of disease time course in ADAD, it may facilitate cross‐disease comparisons with other forms of AD where EYO is not available, particularly in participants who are in the preclinical phase of AD.

## 2 METHODS

### 2.1 Participants and study design

**RESEARCH IN CONTEXT**

1. **Systematic review**: The authors reviewed the literature using PubMed, searching for “sampled iterative local approximation” (SILA), “amyloid chronicity,” and “amyloid time.” SILA has been validated in sporadic AD and applied in Down syndrome AD, but it has not been considered in ADAD, where biomarker elevation and dementia appear in a predictable manner based on genetic information
2. **Interpretation**: For comparisons across broad forms of AD, anchoring analyses to pathology progression – using SILA or similar amyloid‐based approaches – may have utility for studying disease progression relative to the putative start of preclinical AD. Variability in the timing of cognitive changes following the development of amyloid pathology suggests these approaches have less utility near symptom onset.
3. **Future directions**: SILA can quantify heterogeneity in preclinical disease duration. Future investigations of the relationship between ‐omics data and estimated preclinical disease duration could yield valuable insights into mechanisms behind advancement from early pathology to observable clinical symptoms.

The Dominantly Inherited Alzheimer Network (DIAN) observational study is a multisite international research consortium that recruits individuals from families with ADAD mutations. Participants were included in this study if they were carriers of an autosomal dominant mutation (see *Genetics*) and had completed longitudinal amyloid PET. Because of known differences in amyloid PET binding patterns *APP* Dutch mutation carriers^21^ and a previously identified escapee^22^ were excluded from analysis. Written consent was obtained for all participants, and study protocols were approved by local Institutional Review Boards of all DIAN sites.

### 2.2 Genetics

DNA samples were collected at enrollment and genotyped using a TaqMan assay (Applied Biosystems, Waltham, MA, USA). Pathogenic variations in either *APP*, *PSEN1*, or *PSEN2* were identified in these carriers of autosomal AD. Individuals with the *PSEN1* mutation were further stratified by location of mutation, as either before or after codon 200^23^ as well as on either transmembrane or cytoplasmic domains.^24^ Individuals were classified as *APOE* ε4‐positive if they had at least one copy of the ε4 allele, including *APOE* ε2/ε4.

### 2.3 Estimated age at symptom onset

Age at symptom onset is evaluated preferentially: If an individual has had demonstrated cognitive decline (CDR > 0), the estimated age of decline is used (Supporting Information); if decline has not been observed, the mean onset age for individuals with the same mutation is used; if neither of these estimates is available, the individual's parental age of onset will be used to estimate the individual's age at symptom onset. EYO is calculated by subtracting the observed or estimated age at which the individual will develop symptoms based on family/genetic history (e.g., 40 years) from the participant's current age (e.g., 30 years, EYO −10 years).

### 2.4 Calculation of cognitive composite

The DIAN cognitive composite consists of the Trail Making Test Part B, the category fluency test (Animals), the Logical Memory delayed recall score from the Wechsler Memory Scale‐Revised, and the Digit Symbol Coding test total score from the Wechsler Adult Intelligence Scale‐Revised.^25^ We calculated this cognitive composite by Z‐scoring the performance of the mutation carriers relative to the baseline visit scores of all cognitively unimpaired mutation non‐carriers. We calculated the composite as the average of these Z‐scores, as long as one or fewer test scores were missing for the clinical visit. All longitudinal visits were used.

### 2.5 Neuroimaging

Magnetic resonance (MR) images were obtained on 3T Siemens or GE scanners. Reconstructed T1‐weighted MR images were region of interest‐segmented (FreeSurfer 5.3, Desikan‐Killiany atlas) and used for anatomical reference for PET. Dynamic amyloid PET was obtained using ^11^C‐Pittsburgh Compound B (PiB).^26^, ^27^ Reconstructed PET time‐series images were processed using the PET Unified Pipeline (PUP, https://github.com/ysu001/PUP).^28^ Briefly, images were smoothed to achieve a spatial resolution of 8 mm^28^ and corrected for interframe motion using in‐house software.^29^ The standardized uptake value ratio (SUVR) in each region was calculated with cerebellar gray as a reference region using the 40‐ to 70‐min post‐injection window.^23^ A summary value for cortical amyloid^30^ was calculated as the arithmetic mean of the partial volume corrected (regional spread function^28^) SUVR of the precuneus and the superior frontal, rostral middle frontal, lateral orbitofrontal medial orbitofrontal, superior temporal, and middle temporal regions.^31^ SUVR summary values were transformed to Centiloids (CL) using previously published methods^26^, ^32^ for reporting purposes. Individuals were considered amyloid positive (A+) if their cortical summary value exceeded the previously established 1.42 SUVR (16.4 CL).^32^

### 2.6 Sampled iterative local approximation

Sampled iterative local approximation (SILA) is an open source, publicly available method designed to estimate time from A+ based on longitudinal amyloid PET data (https://github.com/Betthauser‐Neuro‐Lab/SILA‐AD‐Biomarker).^11^ SILA first models the longitudinal trajectory of amyloid accumulation across the sample by discretely sampling amyloid annualized change versus amyloid level at the reference scan, smoothing this curve, and then integrating with Euler's method. The resulting relative time axis is then adjusted with the initial condition that zero time corresponds to the A+ threshold. For each participant, A+ age was estimated by solving the integrated curve for time given an input SUVR value at a reference scan and then subtracting estimated A+ time from age at the reference scan. For individuals who became A+, we used the first observed A+ scan. For amyloid‐negative (A−) individuals, we used their last scan as the reference scan.

### 2.7 Statistical analysis

Quantification of a biomarker outside of the range of detectability can lead to noise. Specifically, in the case of amyloid PET, a minimum density of amyloid plaque is necessary to achieve a suitable signal‐to‐noise ratio. Below this threshold, quantification results predominantly reflect free, non‐specifically bound radiotracer. Therefore, we adapted previously published methods to identify individuals above the minimum threshold for meaningful cortical amyloid PET uptake, based on the assumption that these individuals were likely to accumulate amyloid on future visits.^33^ Briefly, we estimated cortical amyloid annualized rate of change (ARC) using individual‐level linear regression of longitudinal PiB PET data. We applied Gaussian mixture modeling to identify two ARC distributions, with the 99th percentile of the lower distribution set as the threshold for reliable accumulation.^33^ We then performed a cutpoint analysis, optimizing on F1 score, to determine the baseline cortical amyloid PET uptake that best identified reliable accumulators. Precision‐recall analysis, quantified by the F1 score, is preferable when dealing with imbalanced data classes and has been applied to define reliable accumulation.^33^ To better understand the characteristics of the individuals with amyloid PET accumulation below the minimum threshold for reliable accumulation, we examined the distribution of baseline EYOs for these participants. We then performed a test of proportions to assess whether the relative prevalence of the three genetic mutations (*APP*, *PSEN1*, and *PSEN2*) was similar to the prevalence of genetic mutations in the overall cohort of participants.

Next, we estimated time from A+ using SILA.^11^ We limited SILA application to only individuals with longitudinal amyloid PET data exceeding the minimum threshold for reliable accumulation to minimize the risk of spurious estimates of time to A+. The results of the application of SILA to all available longitudinal data are presented in the supplement. We performed an additional sensitivity study on the reliable accumulators in order to evaluate the robustness of the SILA results. We deliberately held out the 28 known amyloid converters (that is, the individuals who transitioned from A− to A+ during the study) and generated the SILA‐based estimates of time from A+ using the remaining data, then applied the model to estimate the age at A+ for the 28 converters.

### 2.8 Characterization of SILA‐estimated age at A+

We estimated age at A+ by subtracting time from positivity from the participant's scan age. For individuals who converted from A− to A+ during enrollment, we used linear interpolation to estimate their conversion age based on the lag between their last A− and first A+ visits. We compared estimated age at A+ from SILA to the (interpolated) observed age at amyloid positivity using linear regression with bootstrapped confidence intervals (1000 iterations), with forecasted age at A+ as the dependent variable and participant sex as a covariate of non‐interest. We calculated mean absolute error (MAE) to quantify the average error in estimate for both this primary analysis and the sensitivity study, which is presented in the supplement.

To increase the number of individuals with observed ages at A+ beyond those who were known to convert from A− to A+ during the study, we also evaluated cases where individuals were either A+ throughout the study or A− throughout the study. In the cases of individuals who were A− throughout the study, we checked whether or not their predicted age at A+ was greater than their final age of participation, as individuals who were A− throughout participation in the study should have SILA‐forecasted ages at A+ that are greater than their maximum age. Similarly, in the cases of individuals who were A+ throughout the study, we checked whether or not their predicted age at A+ was less than their study age at enrollment. Using chi‐squared tests for categorical variables and ANOVA for continuous variables, we compared participant demographics between the individuals who were correctly and incorrectly classified to look for patterns in inaccurate estimations of amyloid chronicity.

### 2.9 Comparison of SILA‐estimated age at A+ and EYO

We compared age at A+ to age at symptom onset using linear regression. We did this first for all participants based on their projected age at symptom onset (*N* = 278) and then limited it to only include participants who had converted to symptomatic AD (*N* = 22). We explored genetic influences by stratifying SILA results by mutation type (APP, PSEN1, and PSEN2), *APOE* ε4 status, and PSEN1 mutation location (pre‐ vs post‐codon 200) and domain (cytoplasm vs transmembrane). Bootstrapped linear regressions (1000 iterations) tested interactions between mutation type and symptom onset age, and Wilcoxon tests compared A+ and symptom onset ages across mutations (results in supplement).

### 2.10 Comparison of SILA‐estimated age at A+, EYO, and cognition

Finally, we assessed cognitive performance using generalized additive models, with the composite cognitive score as the dependent variable and disease progression markers (EYO and estimated time from A+) as independent variables. We also tested a combined model to determine whether both markers provided additional predictive value. Similarly, we used the CDR‐SB score as the dependent variable and disease progression markers, both individually and combined, as independent variables.

## 3 RESULTS

Longitudinal PET‐PiB scans were completed by 379 ADAD mutation carriers (Table S1). The median number of scans completed was 2 (*μ* = 2.66, *ơ* = 0.98). Twenty‐two participants converted from asymptomatic to symptomatic during enrollment, and another 28 converted from A− to A+ during enrollment.

When we calculated the threshold for reliable accumulation, we observed that the 99th percentile for the lower distribution of ARC was 0.052 SUVR/year (approximately 2.3 CL/year) (Figures S1A,B). This rate of accumulation was associated with a baseline cortical amyloid PET uptake of 1.16 SUVR (4.8 CL) (sensitivity = 0.843, specificity = 0.720, and F1 score = 0.685) (Figure S1C). One hundred one participants had baseline scans below 1.16 SUVR. Of these 101 participants, 77% were more than 10 years prior to symptom onset, suggesting that these individuals were enrolled prior to the onset of preclinical AD pathology. Their amyloid PET uptake is likely to increase at a future time. The remaining 23% of participants were within 10 years of symptom onset (median = −3.9 years, range = −9.0 years, 5.1 years). We compared the proportion of genetic mutation prevalence, finding that 64% of these participants were APP mutation carriers. This proportion was significantly greater *(p *= 0.0008) than the observed 13% prevalence of APP mutation carriers in the full cohort of DIAN participants with longitudinal PiB PET.

**FIGURE 1:**
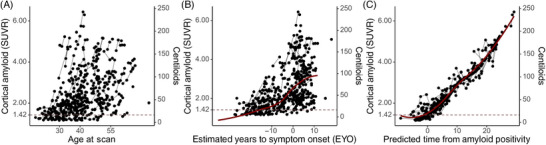
(A) Cortical amyloid PET uptake increases across the lifespan for individuals with autosomal dominant Alzheimer's disease (ADAD). (B) This pathological accumulation occurs prior to symptom onset but has a high degree of variability when it increases relative to estimated years to symptom onset (EYO). (C) Cortical amyloid positron emission tomography uptake can be translated into a chronological estimate of time from amyloid positivity (A+) using the sampled iterative local approximation. The threshold for A+ is shown by a dashed line on all figures.

Application of the SILA model to longitudinal amyloid PET data yielded similar results, whether or not the low amyloid participants (SUVR < 1.163) were included (Figure 1, Figures S2–4). Notably, only data containing reliable accumulators (*N* = 278, Table 1) yielded a minimum A+ time of 9.3 years prior to A+ as compared to the estimate of 50.0 years prior to A+ yielded in the full cohort. The issue is inherent in modeling disease progression in individuals with low amyloid uptake wherein the mean rate of change approaches zero (i.e., people are not accumulating amyloid) and is below a meaningful threshold of amyloid detection by PET. This is most apparent when we consider the estimated number of years that elapse between estimated A+ age and age at symptom onset. In the reliable accumulator subset, more than 50% of individuals develop symptoms between 5 and 18 years after becoming A+, a range we consider reasonable based on published descriptions of the preclinical disease phase in ADAD^5^, ^6^, ^7^, ^20^ (Figure S3B). In contrast, when the model is applied to the entire cohort, more than 50% of individuals become symptomatic between 1 and 16 years after becoming A+, while some individuals become symptomatic as early as 40 years prior to A+.

**TABLE 1:** Participant demographics of individuals with longitudinal amyloid imaging above reliable accumulation threshold.

	Overall	PSEN1	PSEN2	APP	*p*
*N*	278	228	18	32	
Number of visits (mean [SD])	2.69 (1.02)	2.65 (1.00)	3.44 (1.29)	2.56 (0.80)	0.004
Duration of enrollment (mean [SD])	3.13 (2.08)	3.02 (1.93)	5.54 (2.98)	2.59 (1.71)	<0.001
Age at enrollment (mean [SD])	39.87 (10.68)	39.11 (10.53)	41.90 (10.95)	44.10 (10.84)	0.033
Sex (percentage female)	154 (55.4)	130 (57.0)	6 (33.3)	18 (56.2)	0.15
Racial identity (percentage White)	238 (85.6)	190 (83.3)	18 (100.0)	30 (93.8)	0.516
Estimated year of onset at enrollment (mean [SD])	−5.10 (9.61)	−4.96 (9.10)	−10.75 (12.03)	−2.92 (10.76)	0.018
Amyloid cortical PET uptake at enrollment (mean [SD]) [SUVR]	2.36 (1.08)	2.34 (1.07)	2.56 (1.33)	2.43 (1.00)	0.668
*APOE* ε4 carrier (%)	92 (33.1)	72 (31.6)	10 (55.6)	10 (31.2)	0.112
PSEN1 mutation location percentage post‐codon 200 mutation	–	138 (60.5)	–	–	–
PSEN1 mutation domain percentage transmembrane	–	142 (65.7)	–	–	–

Abbreviations: *APOE*, apolipoprotein E; *PSEN*1, Presenilin 1; *PSEN*2, Presenilin 2; APP, amyloid beta precursor protein; PET, positron emission tomography; SD, standard deviation; SUVR, standardized uptake value ratio.

**FIGURE 2:**
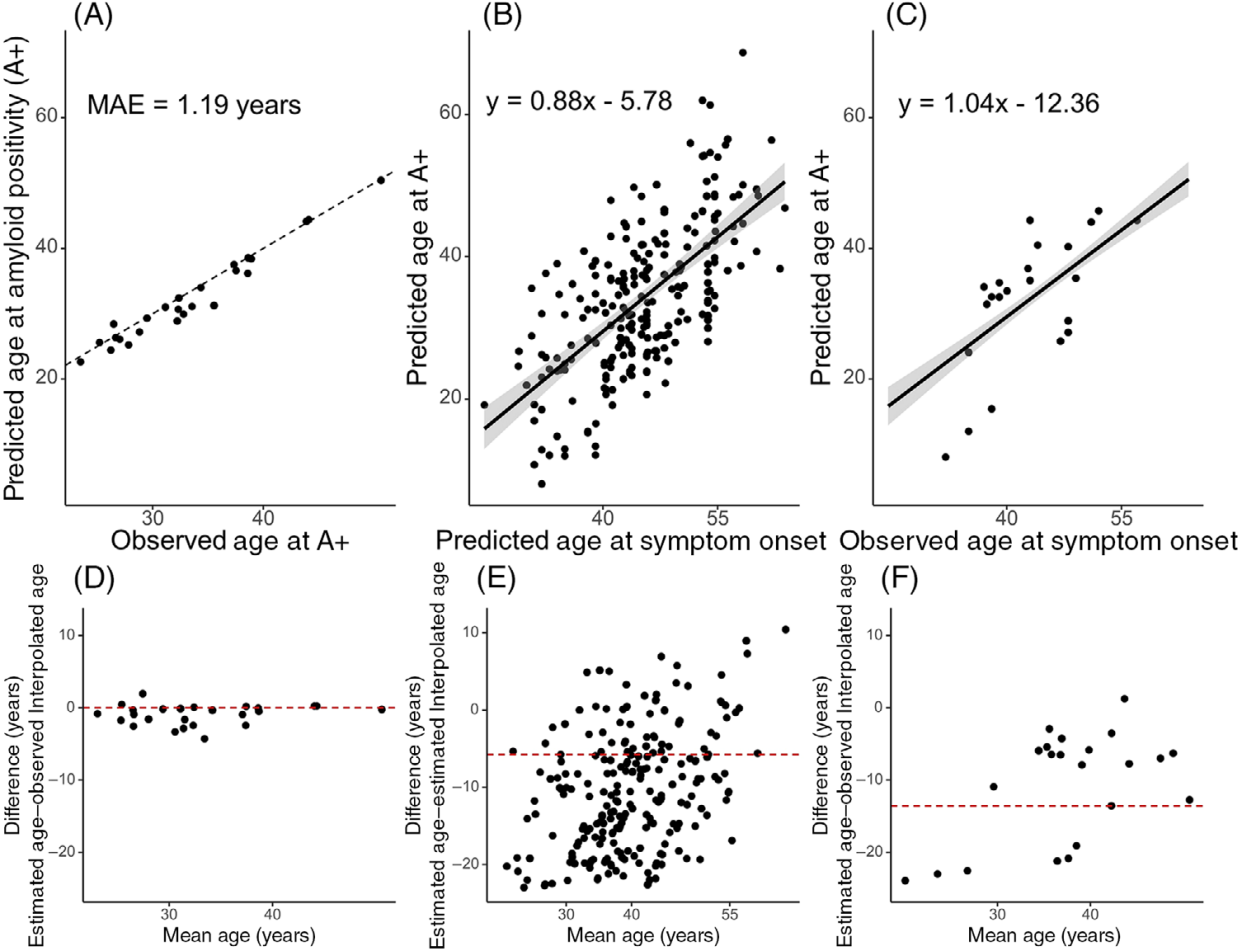
Comparison of sampled iterative local approximation (SILA)‐estimated age at amyloid positivity (A+) to multiple parameters, with corresponding Bland–Altman plots underneath. (A) In individuals who converted from amyloid negative (A−) to A+ during study enrollment (*N* = 28), we observed a mean average error in model prediction less than the typical imaging sampling frequency. Estimates of time at actual conversion of amyloid were based on linear interpolation between the last observed A− and first observed A+ dates. (B) Across the study cohort, using the predicted age at conversion to symptomatic Alzheimer's disease (AD), we observed that the model‐predicted age at A+ explained 39% of the variance in symptom onset. (C) When we limited the cohort to only include participants who converted from asymptomatic to symptomatic AD during study enrollment (*N* = 22), model‐predicted age at A+ explained 45% of the variance in observed age at symptom onset. (D) The Bland–Altman plot highlights systematic underestimation of the SILA‐estimated age at A+ in individuals with younger ages of A+. (E) The *y*‐intercept for the modeled relationship between SILA‐estimated age at A+ and predicted age at symptom onset is shown by a dashed line. A high degree of heterogeneity existed. (F) The *y*‐intercept for the modeled relationship between SILA‐estimated age at A+ and observed age at symptom onset is shown by a dashed line. In younger individuals, A+ and symptom onset occurred more proximately, indicating a shorter preclinical disease duration. In older individuals, A+ and symptom onset occurred farther apart, indicating a longer preclinical disease duration.

When we tested SILA‐estimated A+ age accuracy within the 28 individuals who were observed to convert from A− to A+ during enrollment, we observed a MAE of 1.15 years (Figure 2A). When we excluded these 28 individuals from the model generation and only applied SILA to individuals who remained either A+ or A− throughout, we still observed a MAE less than the sampling frequency of DIAN (approximately one amyloid PET scan every 3 years), although it was marginally higher than the model that included the 28 individuals in the training data (MAE = 2.66 years) (Figure S5). When we compared the age at the conclusion of participation for all A− participants, we found that 56 out of 62 participants had a predicted age at A+ that was greater than their age at the conclusion of the study, which indicates 90.3% success in identifying individuals who were A− throughout participation. When we performed a similar exercise to the participants who were A+ at enrollment, we found that 181 out of 198 A+ participants had a predicted age at A+ that was less than their age at enrollment of the study, which corresponds to 91.4% success.

Evaluation of participant demographics revealed that the six participants who were A− but assigned an age at A+ that was less than their age at the conclusion of the study were on average younger (mean age = 34.7 compared to 38.4 years old at enrollment), but not significantly so (*p* = 0.470). None of the six participants who remained A− throughout the study but were assigned an age at A+ within the timeframe of the study were post‐codon‐200 mutation carriers (compared to 82% of the PS1 mutation carriers [*N* = 17] who were correctly assigned an age at A+ that was greater than their age at the conclusion of the study (*p* = 0.001), which may be consistent with the notion that post codon‐200 mutation carriers accumulate amyloid relatively slowly.^34^ Of the 17 A+ participants who incorrectly had a predicted age at A+ that occurred after study enrollment, they were younger (mean age = 32.0 compared to 43.7 years for the correctly identified A+ individuals, *p* < 0.001), had a lower mean cortical amyloid load (*p* < 0.001), and were earlier in disease progression relative to symptom onset (EYO = −13.4 compared to EYO = −0.72 years for the correctly identified A+ individuals, *p* < 0.001). This suggests that these incorrectly identified individuals may have a more rapid rate of amyloid accumulation than the group‐averaged SILA model, which would be consistent with the idea that pathological accumulation is more aggressive in younger individuals.^35^

Next, we evaluated the relationship between amyloid onset and symptom onset by comparing SILA‐estimated age at A+ to the EYO‐forecasted age at symptom onset (Figure 2B,C). When applied to the entire cohort, there was a significant association between age at A+ and age at symptom onset (*β *= 0.8828, 95% confidence interval [CI]:  0.7541 to 1.021). When we limited our comparison to individuals who had converted to symptomatic AD (*N* = 22), the association increased in strength (*β *= 1.041, 95% CI:  0.5111 to 1.513).

Subsequent analyses tested whether considering genetic information would enhance the accuracy of estimated A+ age (Figures S6–9). The relationship between estimated A+ age and age at symptom onset varied by both overall genetic mutation and location of mutation. It appeared that individuals with the APP mutation may accumulate amyloid pathology more slowly than individuals with the PSEN1 or PSEN2 mutations. The average age at A+ was 33.9 years for PSEN1 mutation carriers, 37.9 (95% CI: 33.7 to 42.0) years for APP carriers, and 35.9 (95% CI: 31.8 to 41.4) years for PSEN2 carriers. Although the relationship between cortical amyloid and time was consistent regardless of location of mutation for PSEN1 carriers (pre‐ vs post‐codon 200), individuals with mutation occurring before codon 200 had significantly younger ages of both A+ (31.1 years compared to 35.2, 95% CI: 32.3 to 38.2 years for post‐codon 200 mutation carriers) and symptom onset (41.2 years compared to 46.0, 95% CI: 43.9 to 48.1 years for post‐codon 200 mutation carriers). We did not observe differences by *APOE*ε4 allele presence or mutation domain. In all stratifications, we were constrained by the number of samples available and have included further discussion of the results in the supplement.

**FIGURE 3:**
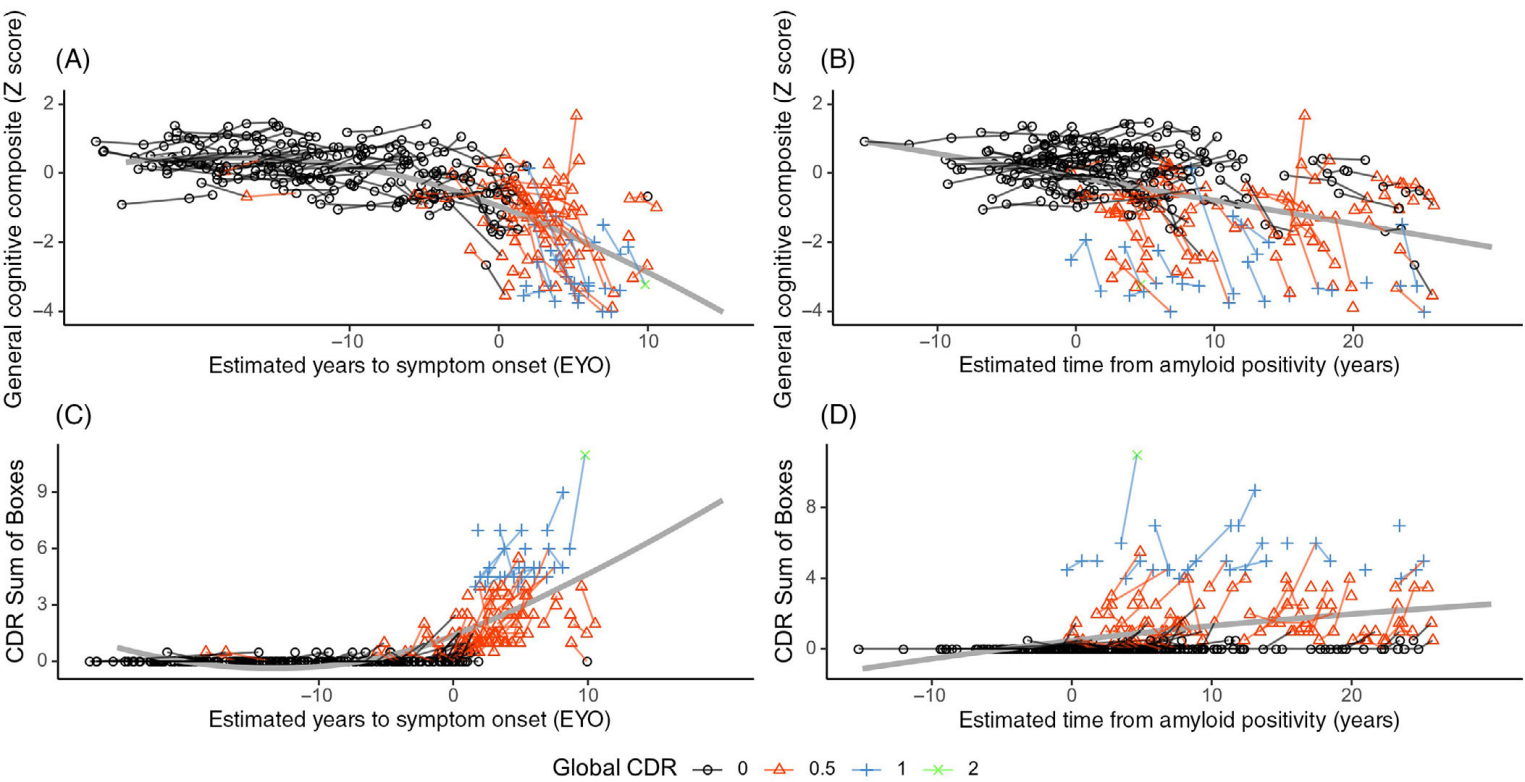
(A) Performance on a general cognitive composite reliably declined around the time of symptom onset in individuals with autosomal dominant Alzheimer's disease (ADAD). (B) Cognitive performance also declined after individuals converted to amyloid positive; however, there was a greater degree of heterogeneity. (C) Clinical Dementia Rating (CDR) Sum of Boxes (SB) scores increase at time of symptom onset in individuals with ADAD. (D) CDR‐SB also increased after individuals converted to amyloid positive; however, there is a substantial amount of heterogeneity.

EYO explained 43% of the variance in performance on the general cognitive composite (Figure 3A), while estimated A+ time explained only 19% (Figure 3B). In both cases the relationship was statistically significant (*p *< 0.001). The effective degrees of freedom (EDF) for the spline fit of the relationship between the cognitive composite and EYO was much higher (EDF = 1.98) than the relationship between the cognitive composite and time from A+ (EDF = 1.00), indicating that a non‐linear fit was merited for EYO but not estimated A+ time. When we evaluated the combined model, EYO continued to be statistically significant (EDF = 1.98, *p *< 0.001) but A+ time was not (EDF = 1.25, *p *= 0.509), suggesting that A+ time did not contribute additional information beyond EYO in ADAD. Results were highly similar when analysis was performed with CDR‐SB as the response variable. EYO explained 57% of the variance in CDR‐SB score (Figure 3C), while estimated A+ time explained only 14% (Figure 3D). For both models, the relationship was statistically significant (*p *< 0.001). The EDF for the relationship between CDR‐SB and EYO was marginally higher (EDF = 1.99) than the relationship between CDR‐SB and time from A+ (EDF = 1.82). When we evaluated the combined model, EYO continued to be significant (EDF = 2.00, *p *< 0.001), but A+ time was not (EDF = 1.34, *p *= 0.802), suggesting that A+ time did not contribute additional information beyond EYO in ADAD.

## 4 DISCUSSION

Our objective was to compare amyloid chronicity as a framework for describing AD progression in ADAD to the established EYO construct. At the group level, amyloid chronicity predicted a 14‐year preclinical disease duration in ADAD, consistent with the AT(N) cascade.^1^ When compared to observed conversion to A+, the error in SILA‐generated estimates was low. However, reasonable estimates for time from A+ could not be generated for all individuals, highlighting that amyloid chronicity modeling cannot be applied to individuals in the absence of biomarker signal. Within this cohort of individuals with ADAD, we observed some degree of heterogeneity, noting particularly aberrant patterns in some individuals carrying the *APP* mutation. Despite this observed heterogeneity, in the majority of cases, the estimated A+ time corresponded to EYO. Although amyloid chronicity predicted cognitive changes, it was not more informative than EYO, highlighting the value of genetic information in ADAD.

Amyloid chronicity and EYO are distinct timescales. EYO predicts future symptom onset based on genetic information,^6^, ^18^, ^19^ while amyloid chronicity is a retrospective estimate of how long an individual has been A+.^11^ Although the model can produce future A+ age estimates for A− participants, our work agrees with prior work^11^ showing *prospective* A+ age estimates are less reliable as reference SUVR values approach the lower limit of amyloid detection by PET imaging. By establishing the initiation of preclinical AD, amyloid chronicity allows for evaluating other biomarkers relative to this starting point. Prior work used amyloid chronicity to compare pathological progression across forms of AD,^36^ and we encourage continued use of this approach, particularly in cases where the pathological changes of interest are temporally closer to the start of the preclinical disease phase than symptom onset.

Our results support amyloid chronicity as a useful timescale for describing amyloid progression, particularly near the time of conversion to A+. For individuals converting to A+ during enrollment, the error in estimating age at A+ was below the study's sampling frequency, regardless of whether or not the converters were included in the training of the model. However, prediction of conversion to symptomatic AD was less accurate, consistent with prior findings that symptom onset varies widely after A+ conversion.^16^, ^17^ Forecasted conversion to symptomatic disease ranged from 10 years prior to 31 years after A+ (half of individuals converted to symptomatic AD between 6 and 18 years after A+), with A+ explaining about 45% of symptom onset variance. This is comparable to findings in sAD (0 to 40 years after A+, 50% symptom onset variance).^9^ For clinical trials (e.g., TRAILBLAZER‐ALZ 3, AHEAD345), this means that utilizing amyloid markers to stage individuals will enrich cohorts for the risk of cognitive decline. However, this result also reinforces the idea that while amyloid chronicity aligns with AD pathology onset, the time between A+ onset and cognitive decline is variable, making amyloid chronicity a less informative predictor of future symptoms compared to EYO. Current models of AD progression suggest that amyloid is insufficient to instigate cognitive decline.^37^ Instead, a combination of both amyloid and tau pathologies are required,^38^ with many other factors including cardiovascular health and educational attainment playing key roles in the degree of clinical impairment that proceeds from these biological measures.^39^

Overall, the current work demonstrates the ability of amyloid chronicity to estimate A+ onset. However, when using such statistical models, some factors must be kept in mind. For any biomarker there is a lower bound of quantification and observations that near this limit are largely dominated by measurement noise. Only once pathology reliably accumulates beyond this noise threshold are biologically meaningful observations obtained. Applying statistical models to the entire range of possible measurements will lead to biologically implausible estimates.^38^, ^39^ For example, observations near the noise floor for PET (e.g., 1.06 SUVR/0 CL) will be predicted by the fitted model to represent implausible estimates (e.g., −45 years, Figure S1). Our work suggests amyloid positivity can only be modestly anticipated (approximately −3 years).

A second caveat is that the generalizability of models is dependent upon the assumption that all individuals follow a relatively similar biological trajectory. Of the individuals excluded from modeling due to low amyloid PET values (*N* = 101), a subset (*N* = 24) were individuals displaying abnormally low amyloid PET uptake relative to their anticipated time of symptom onset. Overall, a disproportionate number of the excluded participants were *APP* mutation carriers.

Investigations into the heterogeneity of ADAD progression have suggested that a myriad of characteristics, including genetic mutation type and location, may impact pathological accumulation.^21^, ^23^, ^26^, ^40^ Lower rates of amyloid accumulation in APP mutation carriers have been reported.^26^, ^40^ Our work seems to support this observation; a disproportionate number of the excluded participants in this modeling exercise were *APP* mutation carriers. Accumulation of amyloid in the basal ganglia has also been noted as a feature in ADAD tied to mutation‐specific features and is also a noted feature in Down syndrome, which features an APP triplication.^41^, ^42^, ^43^, ^44^ Although the chronicity models generally performed very well, such heterogeneity needs to be recognized in order to optimally utilize such measures. Addressing such variability may be particularly critical for applying chronicity approaches to other markers such as tau PET, where there is considerable heterogeneity in both spatial presentation^45^ and rates of accumulation.^35^

Although the initial objective of this investigation was to apply SILA in a cohort of individuals with ADAD and compare its performance with EYO, our understanding of this tool has evolved such that we now also consider it to be a useful approach to defining heterogeneity. The observed heterogeneity in the duration of preclinical AD (as defined by the time elapsed between SILA‐estimated age at A+ and estimated age at symptom onset) highlights potential non‐amyloidogenic pathways in genetically induced AD. It was previously established that amyloid is only a modest predictor of cognition,^16^, ^17^ but future investigations of, perhaps, the relationship between ‐omics‐level data and estimated preclinical disease duration could yield valuable insights into mechanisms behind advancement from early pathology to observable clinical symptoms.

We applied SILA in DIAN, observing reasonable estimates of A+ time and age, for individuals with meaningful cortical amyloid PET uptake. We outlined a data‐driven method to define this threshold based on longitudinal accumulation. Weaker association between A+ time compared to EYO reinforces that amyloid chronicity reflects the start of pathology, but the time from A+ onset to symptoms is variable in ADAD. For comparisons across broad forms of AD, anchoring analyses to pathology progression – using SILA or similar amyloid‐based approaches may have utility for study disease progression relative to the putative start of preclinical AD.

## CONFLICT OF INTEREST STATEMENT

T.L.S.B. has received funding from the NIH and Siemens; has a licensing agreement from Sora Neuroscience but receives no financial compensation; has received honoraria for lectures, presentations, speakers bureaus, or educational events from Biogen, Eisai Genentech, Eli Lilly, Bristol Myers Squibb, Johnson & Johnson, Merck, Medscape, PeerView, Neurology Today, Cedars Sinai Medical Center, Hong Kong Neurological Associationn and the Alzheimer's Association; has served on a scientific advisory board for Biogen; holds a leadership role in other board, society, committee, or advocacy groups for the American Society for Neuroradiology (unpaid) and Quantitative Imaging Biomarkers Alliance (unpaid); and has participated in radiopharmaceuticals and technology transfers with Avid Radiopharmaceuticals, Cerveau, and LMI; she receives research funding from Siemens; she holds two patents on diffusion MR imaging unrelated to this project (US Patent 16/097,457 and US Patent 12,016,701). B.T.C. receives research funding from the NIH. J.H.L. has received research funding from the NIH and NIA. R.J.B. is Director of DIAN‐TU and Principal Investigator of DIAN‐TU001; receives research support from the NIA of the NIH, DIAN‐TU trial pharmaceutical partners (Eli Lilly, F Hoffmann‐La Roche, Janssen, Eisai, Biogen, and Avid Radiopharmaceuticals), the Alzheimer's Association, the GHR Foundation, the DIAN‐TU Pharma Consortium (active members Biogen, Eisai, Eli Lilly, Janssen, and F Hoffmann‐La Roche/Genentech; previous members AbbVie, Amgen, AstraZeneca, Forum, Mithridion, Novartis, Pfizer, Sanofi, and United Neuroscience), the NfL Consortium (F Hoffmann‐La Roche, Biogen, AbbVie, and Bristol Myers Squibb), and the Tau SILK Consortium (Eli Lilly, Biogen, and AbbVie); has been an invited speaker and consultant for AC Immune, F Hoffmann‐La Roche, the Korean Dementia Association, the American Neurological Association, and Janssen; has been a consultant for Amgen, F Hoffmann‐La Roche, and Eisai; and has submitted the US non‐provisional patent application named “Methods for Measuring the Metabolism of CNS Derived Biomolecules In Vivo” and a provisional patent application named “Plasma Based Methods for Detecting CNS Amyloid Deposition.” B.M.A. receives research funding from the NIH and holds a patent (“Markers of Neurotoxicity in CAR T patients”). J.H.R. has received funding from the Korea Dementia Research Project through the Korea Dementia Research Center, funded by the Ministry of Health & Welfare and the Ministry of Science and ICT, South Korea (HU21C0066). D.F.A. receives research funding from the NIH, Roche, and Banner. C.R.S.B. receives research funding through the Hospital Research Foundation Group at the Royal Adelaide Hospital and Queen Elizabeth Hospital. C.C. receives funding from the NIA, the Michael J. Fox Foundation, and the Alzheimer's Association; he has received consulting fees from Circular Genomics and Alector and travel support from Somalogics; he holds both a leadership role and stock/stock options in Circular Genomics. G.W. has received consulting fees from Alector Inc. P.R.M. receives research funding from the BrightFocus Foundation, the Alzheimer's Association, the NIA, and the National Alzheimer's Coordinating Center. I.Y. receives research funding from the Federal Ministry of Education and Research Germany and the German Research Foundation; he has leadership roles with the Neuroimaging Committee of the European Association of Nuclear Medicine, the board of directors of the Brain Imaging Council, Society of Nuclear Medicine and Molecular Imaging, and the Molecular Connectivity Working Group. M.Z. has received honoraria from the Alzheimer's Therapeutic Research Institute and LuMind IDSC. J.J.L.G. receives research funding from the NIA, Alzheimer Association Research Fund, Bright Focus, and the Michael J. Fox Foundation. S.A.S. receives research funding from the NIA. All other authors declare no competing interests. Author disclosures are available in the Supporting Information.

## CONSENT STATEMENT

Written consent was obtained for all participants, and study protocols were approved by local institutional review boards of all DIAN sites.

## Supporting information



Supporting Information

Supporting Information

## Data Availability

The data used in this analysis are available on request to DIAN, provided data request applications are approved by the studies’ committees. Data for DIAN can be requested at https://dian.wustl.edu/for‐investigators/. Open source code for SILA is available at https://github.com/Betthauser‐Neuro‐Lab/SILA‐AD‐Biomarker.
