# Supplementary material for: Comparison of amyloid chronicity and EYO in autosomal dominant Alzheimer's disease
Source: Alzheimers Dement. 2025 Oct 25;21(10):e70812. doi: 10.1002/alz.70812 (PMC12552894; doi:10.1002/alz.70812)
Supplement: Supplementary file 2 — Supporting Information [file ALZ-21-e70812-s002.docx]

**SUPPLEMENT**

**Supplemental Methods on the Definition of Onset Age**

We defined the age of symptom onset (called "decline age") using the Clinical Dementia Rating (CDR), where a score of 0 indicates no symptoms and a score of 0.5 or higher indicates symptom presence. For symptomatic individuals, clinicians estimate the earliest age of symptoms based on standardized criteria, and if multiple estimated ages at observed decline are recorded across visits, we use the average as the final onset age. If a CDR of 0.5 is followed by a return to 0, we treat the 0.5 as a false signal and do not assign an onset age unless symptoms persist. If no decline has been observed, we used the average symptom onset age for individuals with the same genetic mutation, based on a large DIAN database with ~1,500 known cases. If we failed to find a match for genetic mutation, a participant’s parental age of onset was used instead. In the rare case where both parents carried the same mutation, we averaged their onset ages, and if parental data was unavailable, we used the closest affected relative’s onset age instead.

**Supplemental Table 1.** Participant demographics for all participants with longitudinal amyloid PET imaging in the DIAN Datafreeze 17

|  | **Overall** | **PS1** | **PS2** | **APP** | **p** |
| --- | --- | --- | --- | --- | --- |
| N | 379 | 298 | 30 | 51 |  |
| Number of Visits (mean (SD)) | 2.66 (0.98) | 2.60 (0.94) | 3.27 (1.26) | 2.67 (0.89) | 0.002 |
| Duration of Enrollment (mean (SD)) | 3.64 (2.45) | 3.50 (2.37) | 5.32 (2.62) | 3.47 (2.52) | <0.001 |
| Age at Enrollment (mean (SD)) | 37.53 (10.60) | 37.47 (10.26) | 37.49 (11.30) | 37.88 (12.24) | 0.969 |
| Sex (% Female) | 216 (57.0) | 174 (58.4) | 16 (53.3) | 26 (51.0) | 0.562 |
| Racial Identity (% White) | 327 (86.3) | 248 (83.2) | 30 (100.0) | 49 (96.1) | 0.263 |
| Est Year of Onset at Enrollment (mean (SD)) | -9.09 (11.41) | -8.28 (10.77) | -15.03 (12.22) | -10.31 (13.52) | 0.006 |
| Cortical Amyloid PET Uptake at Enrollment (mean (SD)) [SUVR] | 2.01 (1.10) | 2.03 (1.09) | 1.94 (1.28) | 1.92 (1.04) | 0.739 |
| apoe4 Carrier (%) | 126 (33.2) | 92 (30.9) | 16 (53.3) | 18 (35.3) | 0.043 |
| PS1 Mutation Location % Post-codon200 | -- | 186 (62.4) | -- | -- | -- |
| PS1 Mutation Domain % Transmembrane | -- | 182 (65.0) | -- | -- | -- |


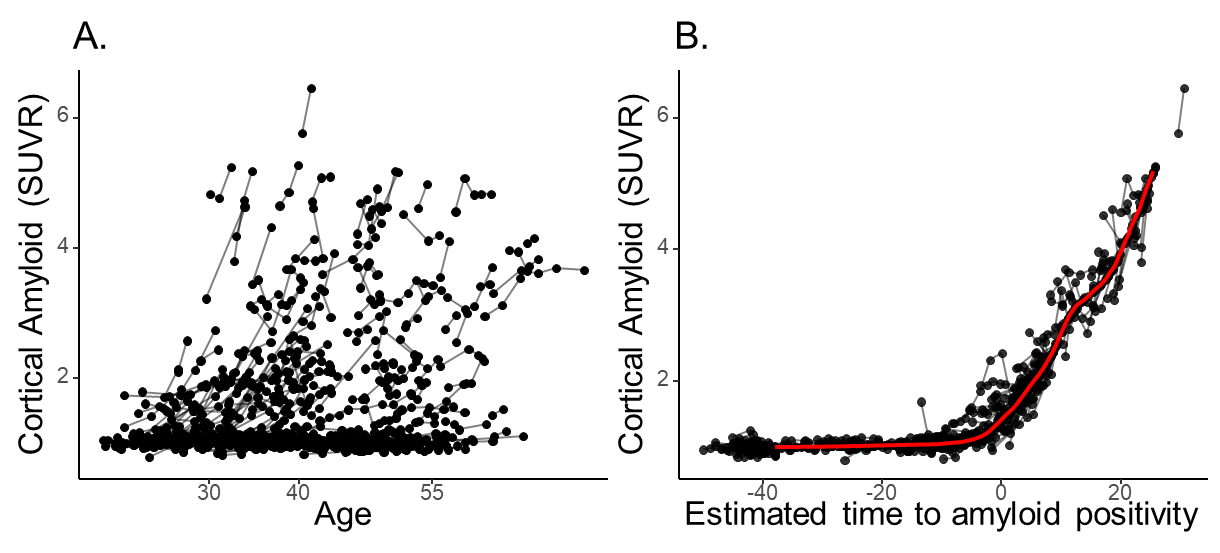


**Supplemental Figure 1.** (A) 379 autosomal dominant Alzheimer Disease mutation-carrying individuals had completed 2 or more amyloid PET scans. (B) Application of SILA to all participants, whether or not they met the cortical amyloid threshold of “reliable accumulation” yielded estimates of up to nearly 50 years prior to amyloid positivity


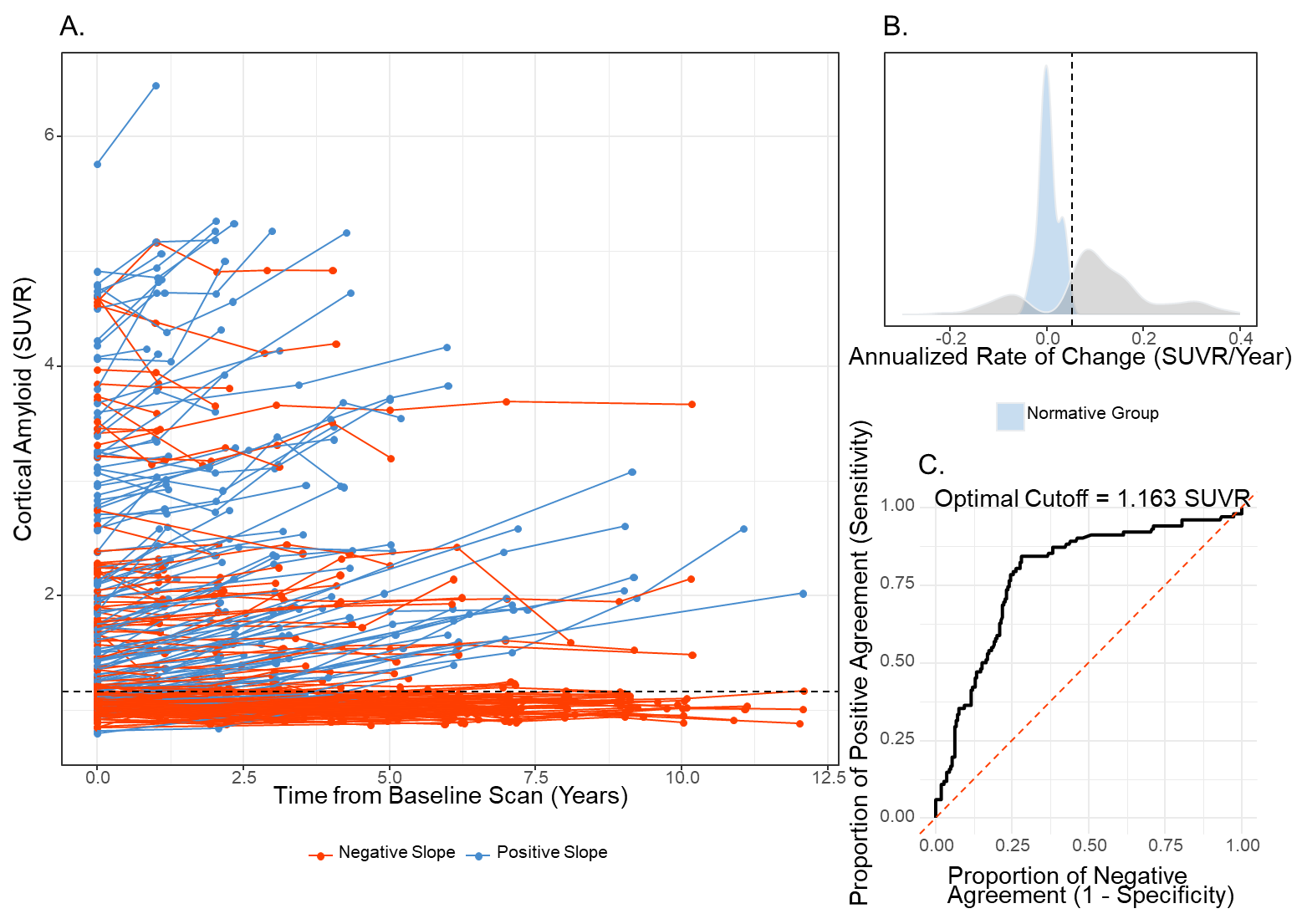


**Supplemental Figure 2.** (A) Longitudinal amyloid data revealed that the majority of participants accumulated amyloid over time (shown in blue). Participants above the threshold of 1.163 SUVR (shown with the dashed line) were more likely to have a positive annualized rate of accumulation. (B) Gaussian mixture modeling reveals that the 99^th^ percentile for annualized rate of change in the normative group was 0.052 SUVR/year. (C) ROC analysis identified that the optimal cutpoint for maximizing the Youden Index to classify individuals as likely to have an annualized rate of change of 0.052 SUVR/year or greater was 1.163 SUVR. Thus, we considered individuals with cortical amyloid PET uptake of 1.163 SUVR or greater to be “reliable accumulators” and were included in the primary results.


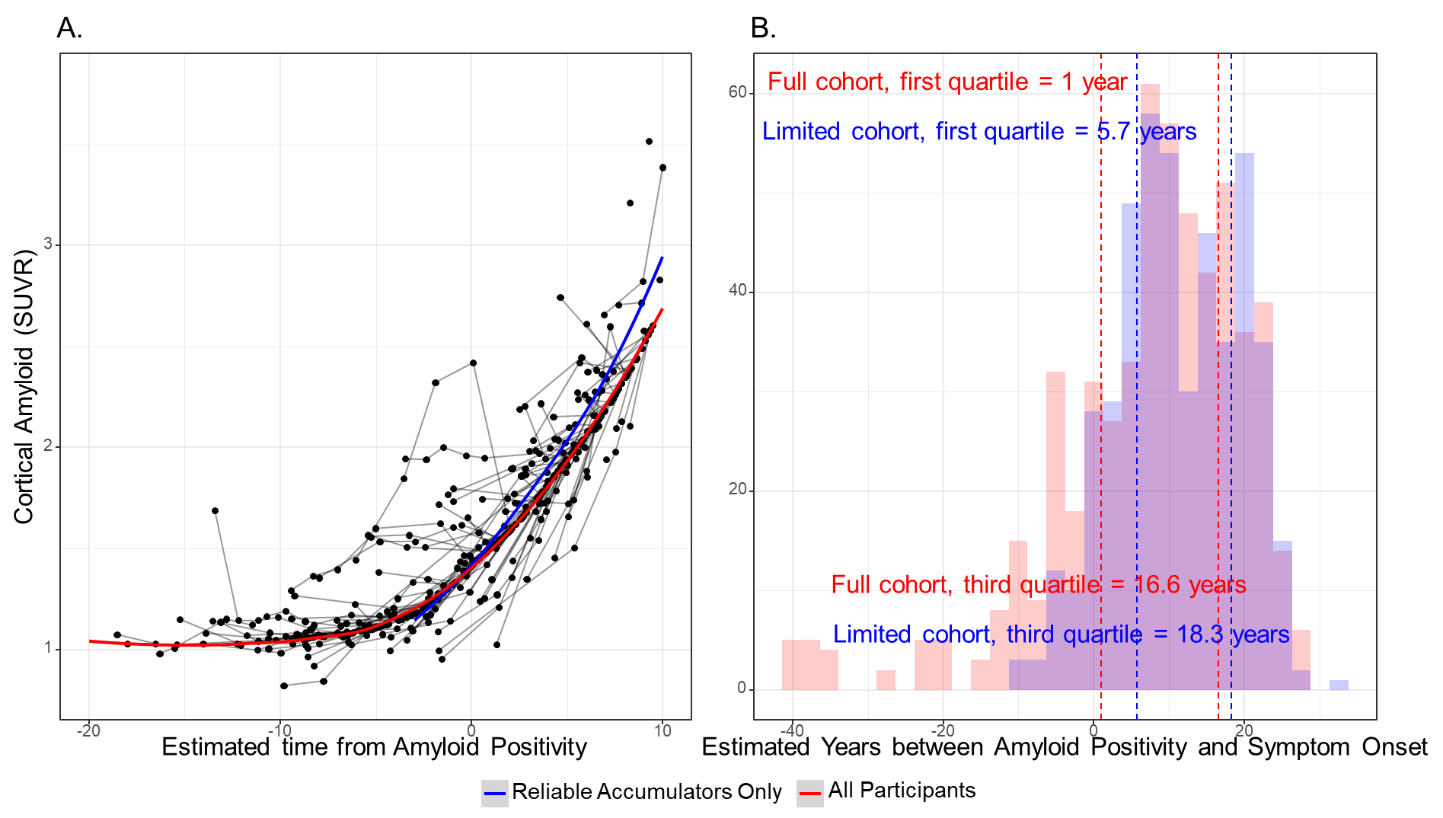


**Supplemental Figure 3.** (A) Estimates of amyloid chronicity from shortly before the threshold for amyloid positivity (A+; 1.42 SUVR) onward were highly consistent whether calculated using only reliable accumulators (shown in blue) or all participants (shown in red). These results highlight that estimates of time from A+ for individuals with a meaningful cortical amyloid PET uptake are not impacted by exclusion of data that is unlikely to contain amyloid PET signal indicative of underlying amyloid pathology. However, inclusion of all individuals without consideration of the minimum detectable signal (i.e., A- participants that have not yet surpassed this limit) for amyloid PET may result in overinterpretation of the data for individuals with low cortical amyloid PET uptake. (B) This is further illustrated when we compare the range of estimated times between when an individual becomes A+ and when symptom onset occurs. When we included individuals with very low cortical PET uptake, we generated estimates that symptom onset occurs as many as 40 years prior to conversion to amyloid positivity, which defies the canonical definition of AD. As a specific example, for the individual who was predicted to become A+ 40 years after symptom onset, the individual was diagnosed as symptomatic roughly a decade prior to their anticipated age at symptom onset. When this individual was scanned 2 years after their symptomatic diagnosis, they had an observed cortical amyloid PET utptake of less than 1 SUVR, suggesting an absence of amyloid plaques. The nonsensical estimate of an individual becoming amyloid positive 40 years after becoming symptomatic is driven by the combination of the extremely low cortical amyloid value and the diagnosis of Alzheimer Disease in the absence of amyloid plaques. This highlights the utility of only including individuals with amyloid levels detectable by PET.


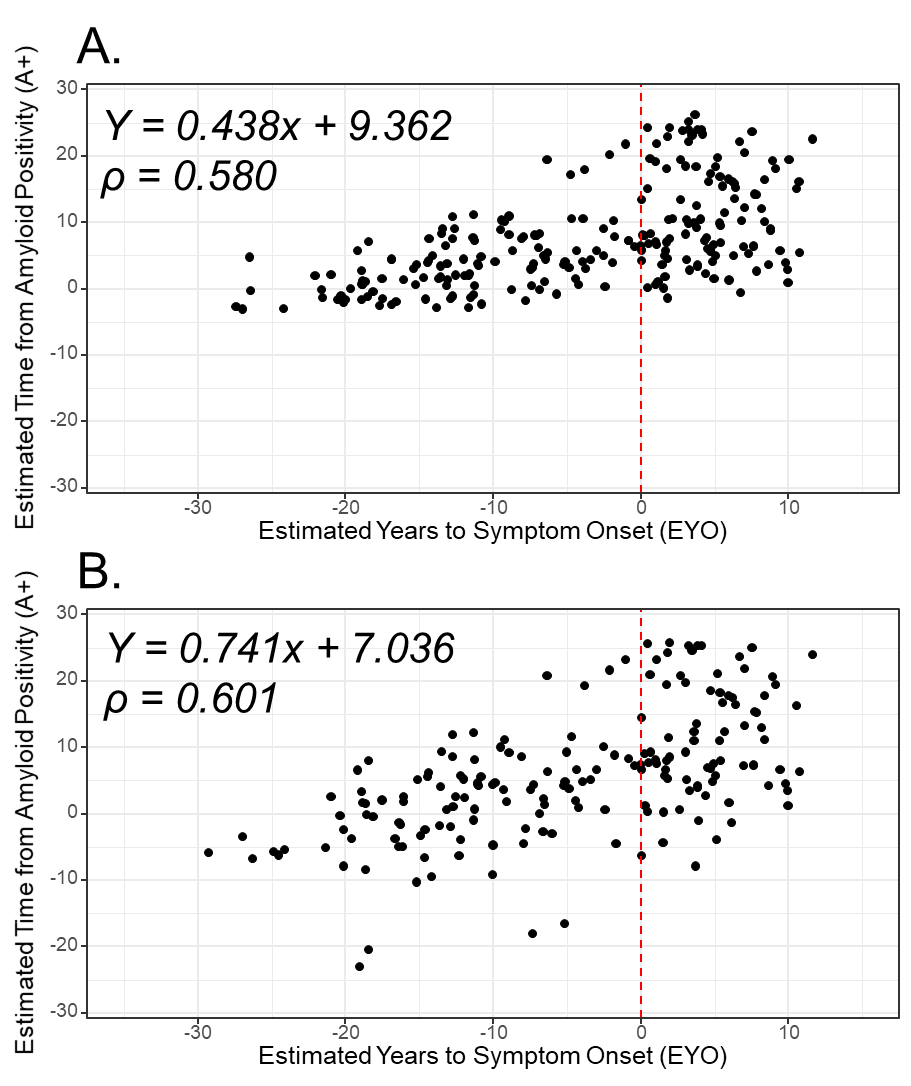


**Supplemental Figure 4.** We present the actual estimates of time from amyloid positivity relative to estimated years to symptom onset (EYO) for the cohort containing only reliable accumulators (A) and for the entire cohort (B). Estimates at the reference scan are displayed. In both cases, there is moderate correlation between amyloid time and EYO, consistent with the results displayed in Figure 2B.

**
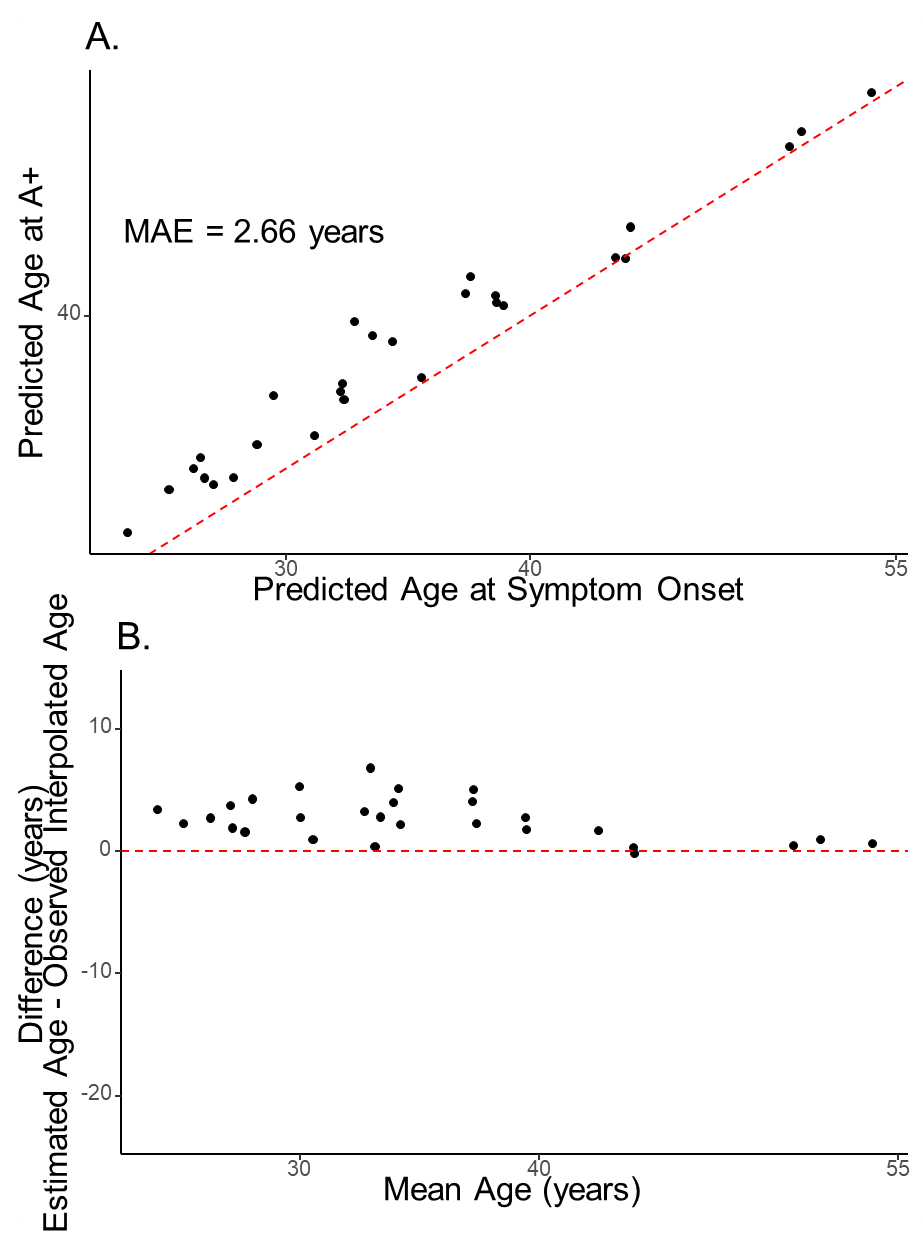
**

**Supplemental Figure 5.** In a sensitivity study we held out the 28 individuals who had a known conversion time from A- to A+. We applied SILA to the remaining participants who were above the reliable accumulator threshold, and then used this defined model to estimate the age at A+ for the participants with the known conversion time. The performance is marginally worse than when the A- to A+ converters were included (MAE = 1.19 years) but still within the typical sampling period for DIAN (3 years) (A). The Bland-Altman plot suggests that in individuals with a younger age of conversion to amyloid positivity, SILA systematically underestimates the estimated age at A+, consistent with results from the model applied to the full cohort (B). When we considered application of SILA stratified by genetic mutation, we observed a qualitatively slower rate of accumulation for individuals with *APP* mutations than either *PSEN1* or *PSEN2* (Supplemental Figure 5A). The relationship between age at amyloid positivity and age at symptom onset was significant (*β_age_ = 0.9210143, 95% CI = 0.7666, 1.0692*), and it was not modified by genetic mutation (*β_APP_ = -18.9387, 95% CI = -48.56664, 9.7877; β_APP*age_ = 0.3853, 95% CI = -0.2053, 0.9867*) (Supplemental Figure 5B). The average age at amyloid positivity was not significantly later for individuals with the APP mutation as compared to the PSEN1 mutation, although symptom onset was (*p = 0.054; p = 0.002*) (Supplemental Figures 5C, 5D). It was not possible to evaluate measures relative to age at symptom onset for individuals with the *PSEN2* mutation because nearly all *PSEN2* mutation carriers included were from the same family and had not yet converted to symptomatic AD, thus they had the same estimated age at symptom onset.


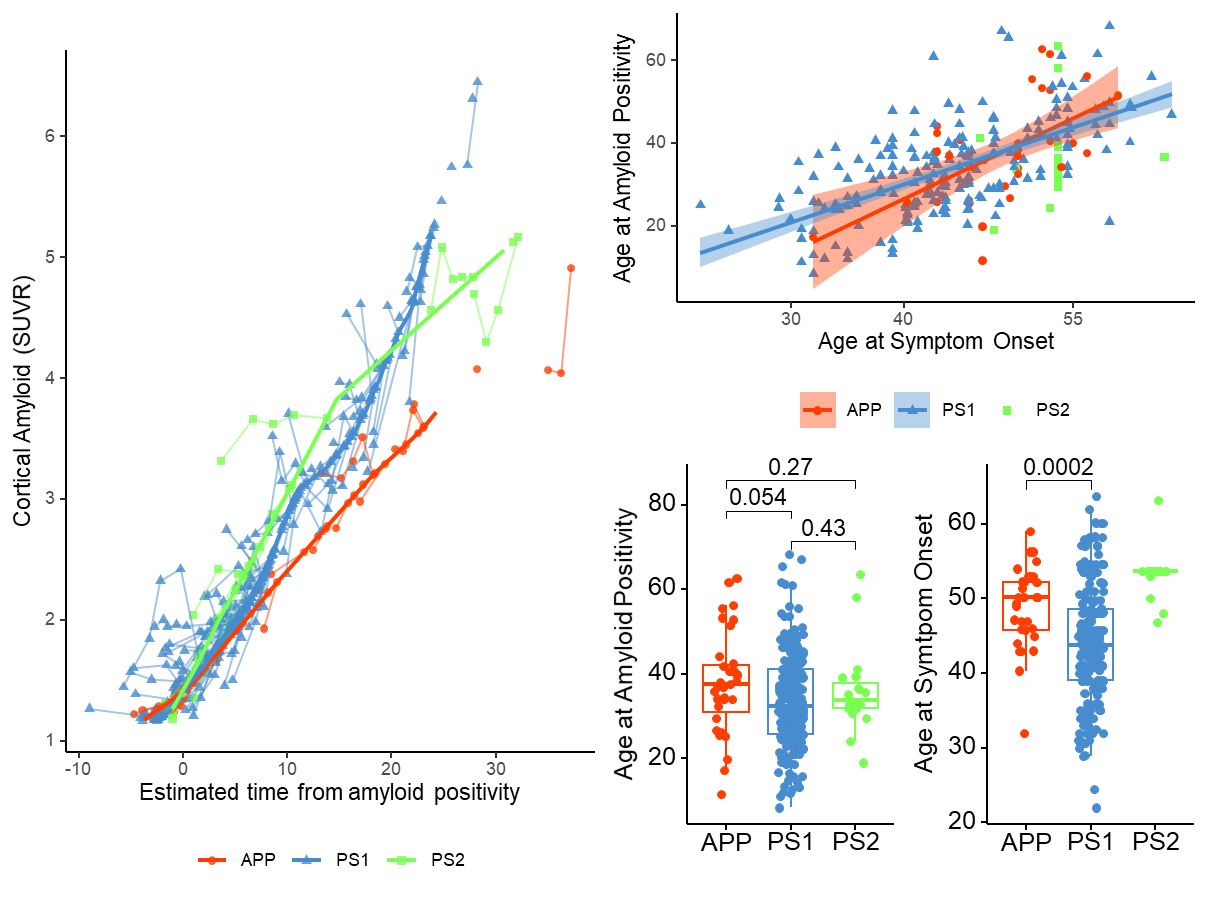


**Supplemental Figure 6**. Comparison of SILA model fit as stratified by genetic mutation. Individuals either had mutations in the Amyloid Precursor Protein (APP), Presenilin 1 (PS1), or Presnilin 2 (PS2). We did not model the relationship between age at amyloid positivity and age at symptom onset for PS2 mutation carriers, as there was insufficient data.

We stratified all participants by whether or not that had at least one copy of the APOEε4 allele, a key risk factor for Alzheimer Disease. When we did this, we did not observe major differences in rate of amyloid accumulation (Supplemental Figure 6A). The relationship between age at amyloid positivity and age at symptom onset was significant (*β_age_ = 0.7709, 95% CI = 0.4651, 1.0985*), but it was not modified by the presence or absence of the APOEε4 allele (*β_APOE4_ = 4.4530, 95% CI = -12.7844, 20.3358; β_APOE4*age_ = -0.0946, 95% CI = -0.4498, 0.2675*) (Supplemental Figure 6B). The difference in age at amyloid positivity or age at symptom onset did not differ significantly by APOEε4 allele (*p = 0.31; p = 0.06*) (Supplemental Figure 6C). The apparent (trend but not significant) elevated age at symptom onset for APOEε4 carriers as compared to non-carriers is in the opposite direction of what we would expect based on the literature, and appears to be driven by one family of APOEε4 non-carriers with extremely pathogenic ADAD (age at symptom onsets occurring before age 30).


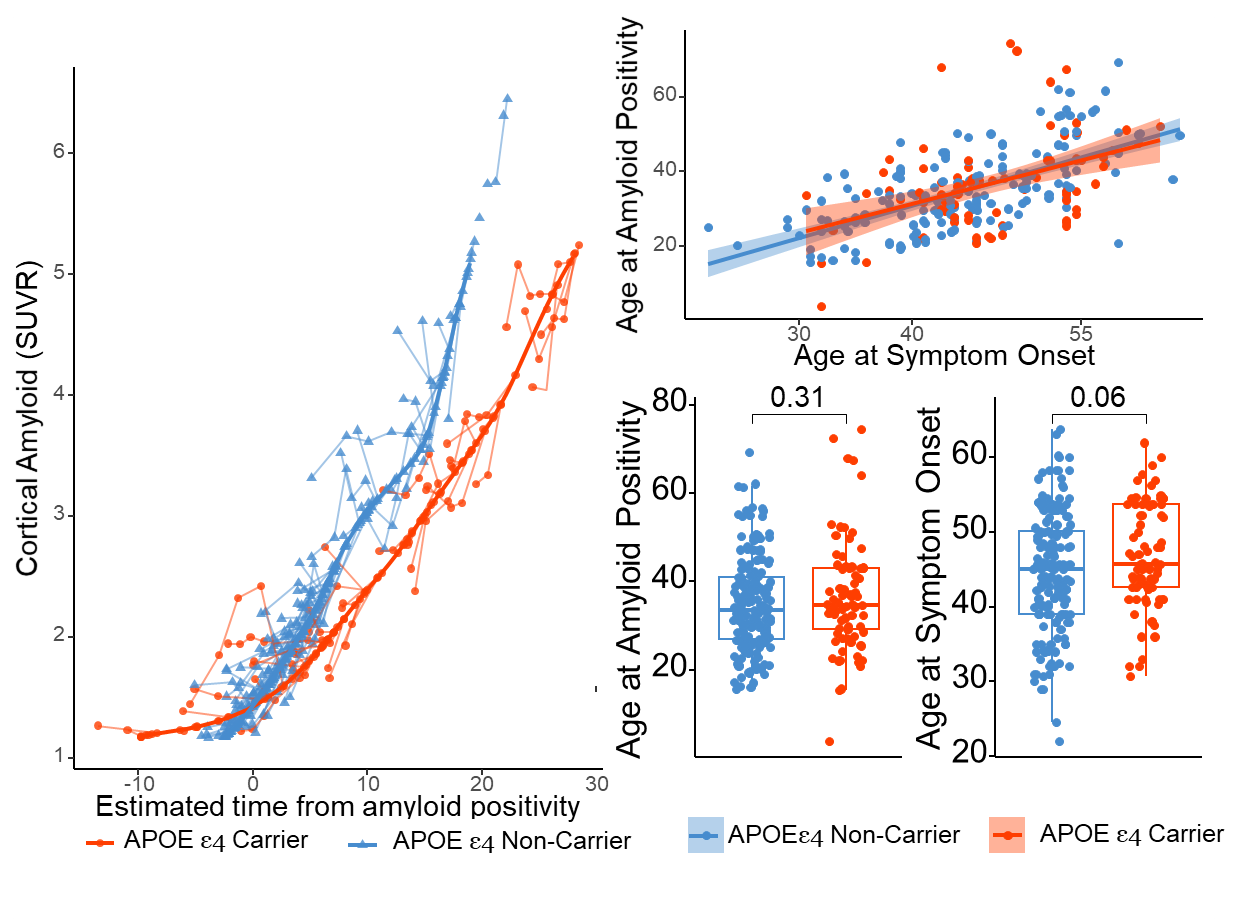


**Supplemental Figure 7**. Comparison of SILA model fit as stratified by presence of the APOEε4 allele.

When we stratified all participants with the Presenilin-1 (*PSEN1*) genetic mutation by the location of the mutation (either pre- or post-codon 200), we observed a qualitatively faster rate of amyloid accumulation for individuals with a pre-codon 200 mutation (Supplemental Figure 7A). Although individuals with a pre-codon 200 mutation appeared to accumulate amyloid faster, there was no difference in the relationship between age at amyloid positivity and age at symptom onset for *PSEN1* carriers, regardless of location of the mutation (*β_Codon_ = 4.2250, 95% CI = -10. 0455, 18.0880; β_Age*Codon_ = -0.0953, 95% CI = -0.4168, 0.2215*), although there was a significant relationship between age at amyloid positivity and age at symptom onset (*β_age_ = 0.8018, 95% CI = 0.5865, 1.0279*). Individuals with mutations located before codon 200 had both younger ages of amyloid positivity (*p = 0.016*) as well as symptom onset (*p < 0.0001*).


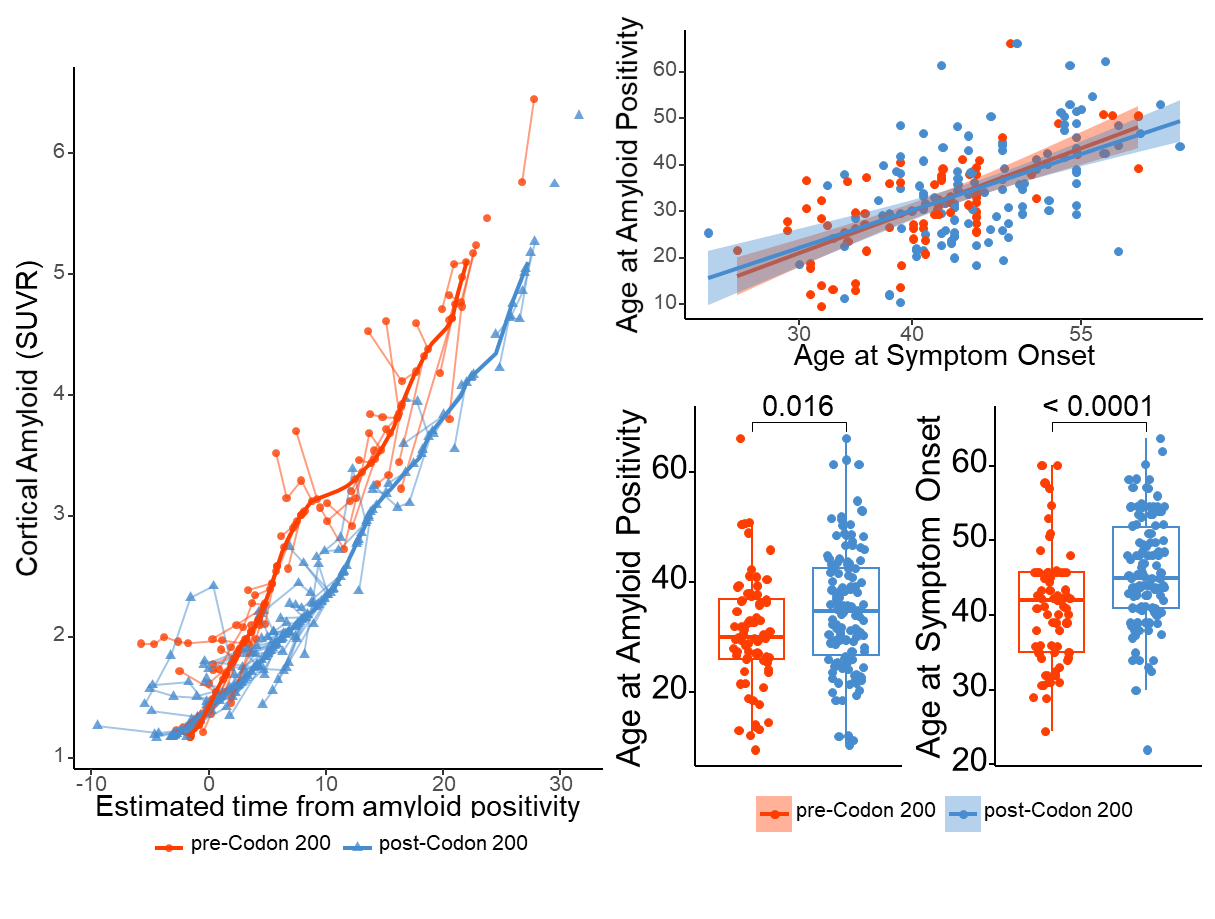


**Supplemental Figure 8**. Comparison of SILA model fit as stratified by location of the mutation on the PS1 gene

We stratified participants with *PSEN1* mutations by the domain of their genetic mutation (cytoplasm or transmembrane). We did not observe major differences in rate of amyloid accumulation (Supplemental Figure 8A). However, the relationship between age at amyloid positivity and age at symptom onset was significant (*β_age_ = 0.6195, 95% CI = 0.3211, 0.8557*). This relationship was modified by domain of the genetic mutation (*β_Domain_ = -17.0834, 95% CI = -31.7068, -4.9349; β_Domain*age_ = 0.3676, 95% CI = 0.0976, 0.7042*) (Supplemental Figure 8B). There was no difference in age at amyloid positivity or age at symptom onset by mutation domain (*p = 0.83; p = 0.66*) (Supplemental Figure 8C). The apparent impact of mutation domain may be driven by the large proportions of participants with familial membership in two specific families, one with a mutation in the cytoplasm domain and a predicted age at symptom onset of 46, and one with a transmembrane mutation and a predicted age at symptom onset of 54.


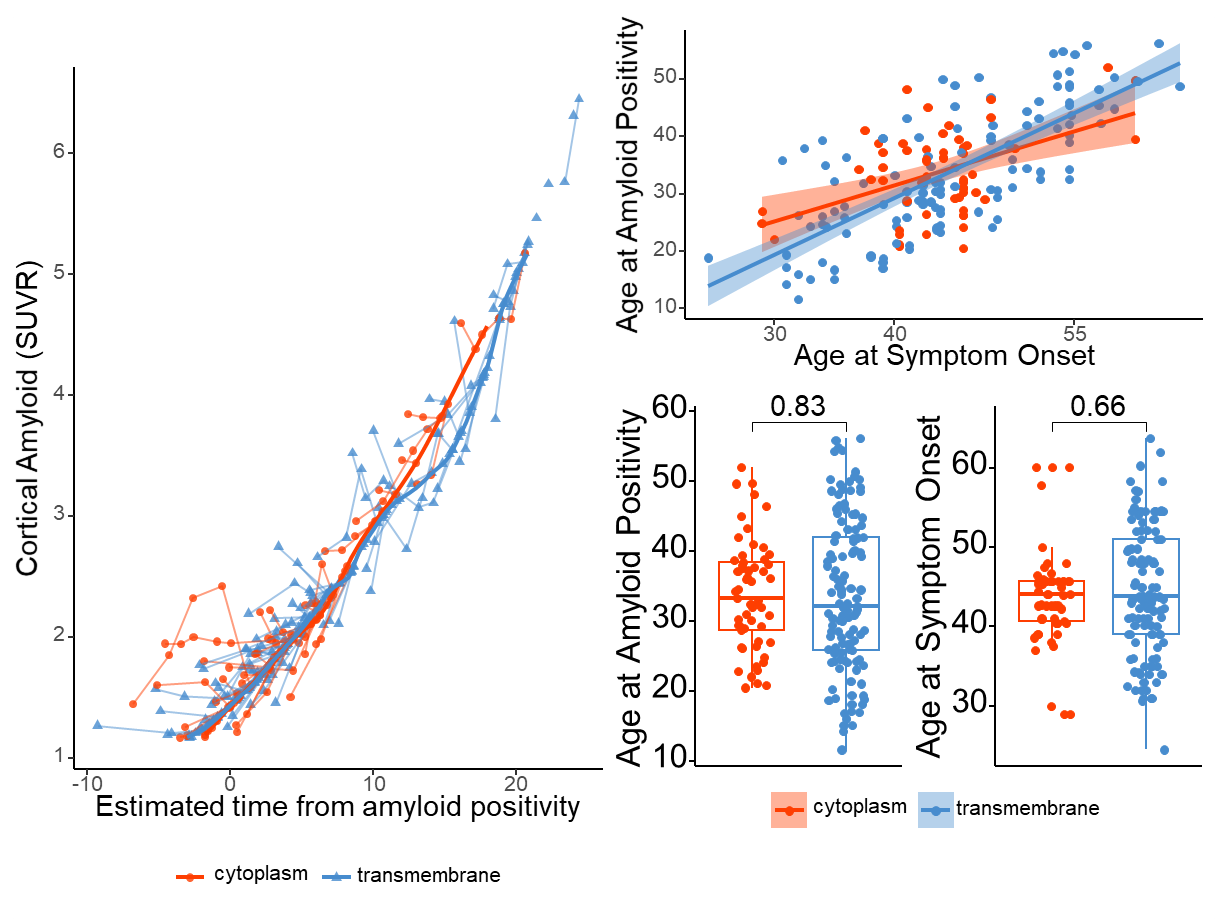


**Supplemental Figure 9**. Comparison of SILA model fit as stratified by domain of the mutation on the PS1 gene
